# Effects of living environment quality on cultural heritage responsibility behaviors of residents in traditional villages: a case study from Hangzhou, China

**DOI:** 10.3389/fpsyg.2025.1600506

**Published:** 2025-12-18

**Authors:** Minglei Jin, Jian Cao, Hangfei Zhao, Zhuojun Li

**Affiliations:** 1 Jing Hengyi School of Education, Hangzhou Normal University, Hangzhou, China; 2 Department of General Education, Tourism College of Zhejiang, Hangzhou, China; 3 Endicott College, Woosong University, Daejeon, Republic of Korea; 4 School of Modern Transportation and Commerce, Tourism College of Zhejiang, Hangzhou, China; 5 Faculty of Creative Tourism and Intelligent Technologies, Macao University of Tourism, Macao, Macao SAR, China

**Keywords:** living environment quality, resident responsibility behaviors, place attachment, pride, sequential mediating effects

## Abstract

**Objective:**

Based on the PAD temperament model, this study aims to systematically reveal the influence path of living environment quality in traditional villages on residents’ cultural heritage responsibility behaviors, and verify the individual mediating effects and sequential mediating effect of place attachment and pride, so as to provide empirical support for the theoretical system of cultural heritage protection.

**Methods:**

A questionnaire survey was conducted among 425 residents from 4 traditional villages in Hangzhou, Zhejiang Province, China to collect data. Core variables were measured using mature scales, and the reliability and validity of the scales were tested through a pilot survey (Cronbach’s *α*=0.958) and CFA. SEM was constructed using SPSS 25.0 and AMOS 24.0, and the Bootstrap method was adopted to verify the mediating effects.

**Results:**

Living environment quality had no direct significant impact on cultural heritage responsibility behaviors (*β* = −0.073, *p* > 0.05), but significantly positively predicted place attachment (*β* = 0.958, *p* < 0.001) and pride (*β* =0.765, *p* < 0.001). Both place attachment and pride significantly positively influenced cultural heritage responsibility behaviors (*β* = 0.471, 0.455; both *p* < 0.001), and place attachment positively predicted pride (*β* = 0.391, *p* < 0.001). The mediating effect test showed that the individual mediating effect of place attachment (Estimation=0.451, 95%CI = [0.240,0.687]), the individual mediating effect of pride (Estimation=0.348, 95%CI = [0.179,0.590]), and the sequential mediating effect (Estimation=0.171, 95%CI = [0.073,0.324]) were all significant, forming a complete mediation model.

**Conclusion:**

This study expands the application boundary of the PAD model in the field of cultural heritage protection in traditional villages, and clarifies the sequential mediating mechanism of “living environment quality → place attachment → pride → cultural heritage responsibility behaviors”. The research conclusions provide a scientific basis for the protection practice of traditional villages, and can indirectly promote the sustainable inheritance of cultural heritage by optimizing the living environment and cultivating residents’ emotional identification.

## 1 Introduction

Historical villages are common in a majority of countries across the world (Kastenholz et al., 2012; Mu and Aimar, 2022; Kim, 2016; Chirieleison et al., 2022; Wibowo and Darsono, 2022). They have a long history, rich cultural heritage, beautiful natural scenery, and unique customs. In China, traditional villages are recognized by the government as excellent historical villages. Traditional villages are recognized for their possession of both tangible and intangible cultural heritage, holding significant historical, cultural, scientific, artistic, social, and economic values (MOHURD, 2012). In China, as of June 2024, the Chinese government has published six lists of traditional villages, totaling 8,171 villages. The concept of traditional villages preserves the historical significance introduced by the ancient village (Chirieleison et al., 2022), while also encapsulating the typicality, representativeness, and continuity of traditional, ethnic, and regional cultures (Su et al., 2022). These villages not only represent important architectural and cultural heritage (Liu et al., 2019; Fu et al., 2021) but also serve as valuable spaces in terms of landscape (Li et al., 2024), ecology (Byun, 2015), culture (Yang, 2023), and tourism (Tang et al., 2023). However, with the rapid urbanization of China (Lin et al., 2021), the rise of rural tourism (Ginting et al., 2024), and the uneven distribution of resources within villages (Liu et al., 2022), the sustainable development of traditional villages has become an increasingly urgent issue.

Residents, as the central component of village communities (Finlay and Haan, 2024), play a crucial role in the sustainability of traditional villages. As the primary occupants of these villages, the material conditions, symbolic features, cultural elements, and behavioral norms of their living spaces collectively shape the cultural memory of the residents. These elements influence their self-perception, behavioral tendencies, and sense of identity (Wheeler, 2014). Traditional villages, as historical witnesses, are not only integral to cultural heritage but also serve as important sites for the everyday lives of rural populations. They embody farming traditions and local cultures, housing rich cultural resources, facilitating the transmission of cultural memory, and reinforcing residents’ cultural identity. The active participation and community consciousness of residents are key drivers of sustainable development in these villages (Talò et al., 2014). Similarly, community engagement plays a pivotal role in the protection of cultural heritage. In China, numerous cultural heritage initiatives have successfully integrated community participation, yielding promising results (Li et al., 2020). Community involvement has thus emerged as a critical strategy for ensuring the sustainable development of cultural heritage in traditional villages (Jing et al., 2024). Despite a growing body of research on the relationship between residents and the sustainable development of cultural heritage, which often focuses on factors such as residents’ attitudes (Giannakopoulou and Kaliampakos, 2016), values (Xi et al., 2021), and behaviors (Jia et al., 2021), studies examining the influence of the quality of the human settlement environment remain scarce. This study addresses this gap by exploring how the quality of the human settlement environment in traditional villages affects residents’ cultural heritage responsibility behaviors.

To better understand the association between multidimensional people–environment interactions and resident behaviors, this research also delves into the mediating factors involved. As a kind of emotional connection, place attachment (PA) involves not only the dependence on the physical environment but also the identification of local history, culture, and social relations. Pride, as a deep emotional experience, involves comprehensive factors, such as individual personality characteristics, mental health, social status structure, and attribution tendency, and is an internal moral consciousness and value concept of individuals (Dickens and Robins, 2022). Place attachment can provide an emotional basis for the generation of pride (Jiao et al., 2023), and as a positive factor, it helps to enhance the pride and sense of belonging of local residents (Ujang and Zakariya, 2015). Currently, few studies integrate place attachment and pride as mediators within a single theoretical framework to explore residents’ cultural heritage responsibility behaviors. Therefore, this study will explore how the quality of the living environment in traditional villages promotes the residents’ place attachment and pride and how it influences the residents’ cultural heritage responsibility behaviors under the PAD temperament model.

This study uses the PAD model to examine the impact of the quality of human settlements on the cultural heritage responsibility behavior of residents in traditional villages, mediated by place attachment and pride. The specific research objectives are (1) to study the formation of individual cultural heritage responsibility behavior within the framework of the PAD model from the perspective of traditional village residents; (2) to explore the impact of the quality of human settlements on the cultural heritage responsibility behavior of residents in traditional villages; and (3) to investigate whether place attachment and pride are enough to mediate the external environment and behavior. By incorporating these two mediating variables, this study aims to gain a deeper understanding of the motivations and mechanisms underlying residents’ cultural heritage responsibility behavior. This approach will provide a clearer exploration of the psychological impact mechanism through which the quality of the living environment influences these behaviors.

The remainder of this article is organized as follows. The second section reviews the existing literature and presents the research hypotheses. The third section provides a detailed analysis of the data, which is followed by empirical results from the data analysis. The fourth section includes discussion, theoretical contributions, and managerial implications. The final section presents the research limitations and directions for future research.

## 2 Theoretical framework and hypotheses development

### 2.1 PAD temperament model

The PAD model is a core psychological framework proposed by Albert Mehrabian and James A. Russell for understanding and measuring emotional responses. This model posits that external environmental stimuli primarily influence an individual’s affective state, which in turn drives behavioral responses (Russell and Mehrabian, 1977). It simplifies complex emotions into three basic and relatively independent dimensions: pleasure–displeasure (P), arousal–non-arousal (A), and dominance–submissiveness (D). The selection of the PAD model as the overarching theoretical framework for this study is based on its robust explanatory power for the “environmental stimuli-affective mediation-behavioral response” pathway, particularly in elucidating the impact of diffuse, holistic stimuli such as the physical environment of traditional villages on residents’ internal experiences and enduring behaviors.

The quality of the human settlement environment encompasses comprehensive elements, including natural ecology, infrastructure, and living conditions. A high-quality human settlement environment directly fosters comfort, safety, and satisfaction, which are essentially pleasurable emotional experiences. Extensive environmental psychology research confirms that improvements in the physical environment are among the most direct means of enhancing individual pleasure (Isen and Reeve, 2005). Therefore, mapping the quality of the human settlement environment to the P dimension has a solid theoretical foundation.

Arousal does not refer to physiological excitement but rather to the state of emotional and cognitive arousal and engagement. Place attachment represents a profound emotional bond between residents and their environment, and this deep connection inherently generates sustained psychological arousal and attention. Pride, as a positive self-conscious emotion, evokes a strong, directed emotional energy and willingness to engage—precisely manifesting as high-level psychological activation—when individuals identify with the cultural values of their community. Therefore, treating these two emotions collectively as manifestations of affective activation (A) captures their intrinsic motivational properties in driving behavior.

The Dominance (D) dimension reflects an individual’s sense of control and efficacy over the environment. Residents’ proactive behaviors, such as participating in cultural heritage preservation, discouraging destructive actions, and learning to inherit knowledge, directly demonstrate their perception that they can and should influence the continuity of cultural heritage, embodying a sense of “control” and “ownership.” Such behaviors transcend passive compliance and represent an active, influential behavioral pattern, aligning closely with the essence of “dominance.”

Although models such as the Theory of Planned Behavior (TPB) and the value–attitude–behavior (VAB) model are highly effective in predicting specific behaviors, they focus more on rational cognition and decision-making processes. This study, however, aims to reveal how the holistic external stimulus of the human settlement environment guides behavior by eliciting spontaneous emotional responses in residents. The core strength of the PAD model lies in its emphasis on emotion as the direct mediating mechanism between environment and behavior, which is highly congruent with the study’s central objective of exploring the “affective pathway.” It better explains how a pleasing environment fosters protective behaviors by evoking people’s love and pride. Therefore, we selected this model as the overarching theoretical framework for the study.

### 2.2 Hypotheses development

#### 2.2.1 Responsibility behavior of traditional village residents for cultural heritage

Traditional villages, as carriers of cultural heritage, hold significant historical, cultural, economic, and social values (Wu et al., 2020). Promoting their sustainable development not only ensures the preservation of tangible and intangible heritage but also fosters residents’ satisfaction and sense of belonging within their living environments (Xu and Wang, 2021). Scholars have approached the protection of traditional villages through various frameworks, such as heritage conservation (Zhou et al., 2019), cultural ecological preservation (Fang and Li, 2022), and landscape protection (Li, 2015). Practical strategies for sustainable growth include production-living-ecology integration (Kong et al., 2021), resilience enhancement (Wang and Zhu, 2022), contiguous protection (Zhao et al., 2024), and digital preservation techniques (Zuo et al., 2023).

Rural tourism, often leveraging the unique cultural and natural assets of traditional villages, has become a key driver of economic growth and sustainability in recent years (Gao and Wu, 2017; Yanan et al., 2024). However, the rapid influx of external influences, including tourists and commercial enterprises, frequently disrupts social harmony, leading to conflicts among stakeholders such as residents, governments, and tourism operators (Yang et al., 2013; Zhu et al., 2025). To mitigate these challenges, it is essential to balance economic development with the preservation of cultural heritage and address the diverse needs of the community. Ensuring residents’ active participation and safeguarding their living environment are critical steps toward fostering harmonious and sustainable development in traditional villages (Weng and Peng, 2014).

Residents’ responsibility for cultural heritage refers to the actions undertaken by individuals, guided by their subjective awareness, that align with legal norms and contribute to the protection and inheritance of cultural heritage. These activities aim to safeguard the integrity, authenticity, and sustainability of cultural heritage and ensure its proper protection and rational use (Gursoy et al., 2019). The evolution of cultural heritage from a focus on material elements to intangible aspects highlights the need to preserve not only historical buildings and artifacts but also traditional skills, arts, and folk customs (Vecco, 2010). As a product of dynamic interactions between humans and nature, cultural heritage serves as a repository of village memory while fostering rural cohesion and revitalization (Li, 2023). Residents, as key stakeholders, play a pivotal role in heritage protection through their attitudes, behaviors, and active participation (Lourenço-Gomes et al., 2019). The sustainability of intangible heritage relies heavily on its engagement, underscoring the necessity of cultivating a strong sense of belonging and identity among community members to ensure its continued preservation (Lenzerini, 2011; Qin and Leung, 2021). In the process of rural heritage protection and management, the enhancement of residents’ emotional attachment and sense of identity to the village can more effectively guide them to actively protect the cultural heritage (Chen and Wang, 2024), thus promoting the sustainable development of traditional villages. Therefore, this study posits the following hypothesis:

*H1:* The quality of the living environment has an impact on the cultural heritage responsibility behavior of residents in traditional villages.

#### 2.2.2 Rural living environment quality

The rural human settlement environment is an integrated system of material and non-material elements essential for the daily life and production activities of rural residents (Hu and Wang, 2020). It encompasses natural conditions, infrastructure, and resources that support livelihoods, work, leisure, and social interactions. Beyond these physical aspects, the environment includes industrial development, public services, environmental satisfaction, living conditions, and community governance, forming the foundation for a well-functioning rural society (Wang et al., 2021). Enhancing the quality of rural human settlements is critical for improving residents’ quality of life and promoting sustainable rural development.

During China’s urbanization process, significant urban–rural disparities emerged, particularly across eastern, central, and western regions (Jiang et al., 2022). To address these imbalances, the government launched the rural revitalization strategy, with the improvement of rural living environment quality being the central focus (Wei et al., 2023). Key initiatives, including the *Three-Year Action Plan on Improving Rural Living Environment* and the *Five-Year Action Plan on Improving Rural Living Environment (2021–2025)*, aim to enhance infrastructure, environmental conditions, and governance. These efforts improve residents’ quality of life and support sustainable development in rural areas by addressing population, land, and industrial needs (Fang et al., 2022; Wang and Zhu, 2023). Furthermore, the interplay between rural living environments and sustainable development—particularly the societal, economic, and cultural benefits of improved environments—has become a key area of focus for future research in this domain (Ma et al., 2016).

The environment has a significant impact on human behavior, habits, and psychological characteristics (Klöckner, 2013). As the carrier of people’s daily living space, the living environment not only includes micro-physical space environment, such as housing buildings, but also includes macro-ecological, physical, and cultural environment, such as climate environment, community facilities, public services, and social policies (Cheng et al., 2024). Both family living environment (Graham et al., 2015) and community living environment (Gallimore et al., 1993) can have an impact on people’s happiness, sense of identity, loneliness, and satisfaction (Prezza et al., 2001). Currently, researchers have established that both the physical and social environments are strongly correlated with place attachment (Friesinger et al., 2022). Therefore, the following hypothesis is proposed in this study:

*H2:* The quality of the living environment affects the place attachment of residents in traditional villages.

There is also a strong relationship between community environment and pride (Renzaho et al., 2012). A high-quality living environment can significantly improve the quality of life of residents, including providing clean air, safe water, convenient transportation, and well-developed public service facilities. These improvements make residents feel more comfortable and convenient in their daily life, thus enhancing their residence satisfaction (Zolnik, 2011). When residents are satisfied with their living environment, they will naturally have pride in the community or city they live in. Therefore, this study posits the following hypothesis:

*H3:* The quality of the living environment affects the pride of residents in traditional villages.

#### 2.2.3 Mediating role of place attachment

Despite the rapid pace of globalization and increasing mobility worldwide, places remain powerful sources of emotional attachment (Lewicka, 2011). Place attachment PAhas become a central topic in environmental psychology, highlighting the psychological bond between individuals and their specific locales (Jorgensen and Stedman, 2001). Although the concept and characteristics of place attachment are expressed differently across various studies, they consistently emphasize the connection people feel toward their environments. The majority of research studies view place primarily as a social environment, with the intensity of place attachment varying across spatial scales and dimensions. Notably, social attachment often outweighs physical attachment in its influence (Hidalgo and Hernandez, 2001).

Furthermore, extant literature suggests that a supportive and conducive living environment can enhance residents’ emotional connection to their communities, thereby promoting greater civic participation (Bayat et al., 2022). Place attachment acts as a mediating factor, linking residents’ emotional ties to their active involvement in community activities (Shin and Yang, 2022). Encouraging such participation is essential for fostering a dynamic community atmosphere that supports the preservation of cultural heritage. Researchers have found that place attachment can play a mediating role in the relationship between natural and human well-being (Basu et al., 2020), community protection and residents’ participation in pro-environmental behavior (Buta et al., 2014), and adolescents’ pro-environmental behavior and happiness (Bartolo et al., 2023). Therefore, this study proposes the following hypotheses:

*H4a:* Place attachment is positively correlated with cultural heritage responsibility behaviors of traditional village residents.

*H4b:* Place attachment plays a mediating role between the quality of the living environment and residents’ cultural heritage responsibility behaviors.

#### 2.2.4 The mediating role of pride

Pride is a complex self-conscious emotion and an intrinsically motivated affect with moral foundations (Van Osch et al., 2018; Tracy and Robins, 2004). As an intrinsic human emotion, pride influences and reinforces behaviors that align with societal norms, supporting the development of an identity consistent with these norms (Bissing-Olson et al., 2016). It plays a crucial role in motivating individuals to adopt sustainable behaviors (Shipley and van Riper, 2022). Among residents, a sense of community pride can be strengthened by improving their quality of life and well-being, which, in turn, fosters the sustainable development of urban cultural heritage (Sadler and Pruett, 2017). In the context of community development, pride acts as both a motivational force and a regulatory factor, thereby encouraging individuals to make sacrifices for collective benefit (Williams and DeSteno, 2008).

Furthermore, studies suggest that pride partially mediates residents’ behaviors within the community, highlighting its role in shaping engagement and cooperation (Cao et al., 2024). Researchers have suggested that pride can foster environmentally beneficial outcomes in certain contexts (Hurst and Sintov, 2022), with pride in environmental behaviors being positively correlated with subsequent engagement in pro-environmental actions. Additionally, pride has been found to predict pro-environmental behavior, particularly when pro-environmental descriptive norms are more favorable.

Although both place attachment and pride belong to the domain of positive emotions, they exhibit clear distinctions in their psychological essences. Place attachment primarily emphasizes the intensity and stability of the emotional bond between individuals and a place, representing an affective connection rooted in belongingness and dependency. In contrast, pride is more focused on a self-evaluative emotion grounded in achievement, value identification, and moral recognition. It stems from the affirmation of one’s own or one’s group’s worth and functions as a more agentic and goal-directed emotional motivation. In this study, improving the quality of the human settlement environment not only enhances residents’ sense of belonging but also activates their pride—an emotion centered on positive self-awareness—by highlighting the cultural uniqueness and value of the community. As an intrinsic moral incentive, pride can directly motivate residents to take responsibility for protecting cultural heritage. Therefore, treating pride as an independent mediating variable holds significant theoretical importance for uncovering the key mechanisms of internal motivation within the “emotion-behavior” pathway. Therefore, this study proposes the following hypotheses:

*H5a:* Pride is positively correlated with cultural heritage responsibility behaviors of residents in traditional villages.

*H5b:* Pride mediates the relationship between the quality of the living environment and residents’ responsibility of cultural heritage.

#### 2.2.5 The sequential mediating effects of place attachment and pride

Numerous studies have demonstrated the intricate relationship between cognition, emotion, and behavior (Gross, 2002; Forgas, 2008), highlighting that individual cognition and emotion significantly influence behavioral outcomes (van Kleef et al., 2017). Emotional states influence attention, memory, judgment, and decision-making, while driving and regulating behavior. Cognitive assessment affects emotional experience, and cognitive reconstruction can change emotion. Cognitive structure guides behavior choice, while cognitive strategies influence the efficiency of these behaviors. Behavioral results provide emotional feedback, and long-term behavioral patterns shape emotional experience. Furthermore, behavior verifies cognitive correctness and prompts individuals to update their cognition to adapt to the new environment. Understanding their relationship helps us to better understand the nature and law of human psychological activities, so as to guide practice and life more effectively. Place attachment, which refers to a cognitive and emotional bond between individuals and their surrounding environment, plays a crucial role in shaping the relational dynamics between people and places.

Research indicates a positive correlation between place attachment, pride, and pro-environmental behavior (Shipley et al., 2023), with pride serving as a mediator between place attachment and individual environmental behavior (Jiao et al., 2023). Building on the established connections between emotion, cognition, and behavior, and the influence of place attachment on pride, this study posits that place attachment and pride sequentially mediate the relationship between the living environment and residents’ cultural heritage responsibility behaviors. Accordingly, the following hypotheses are proposed:

*H6:* Place attachment is positively correlated with pride.

*H7:* Place attachment and pride, in turn, mediate the quality of the living environment and the responsibility behaviors of cultural heritage.

**Figure 1:**
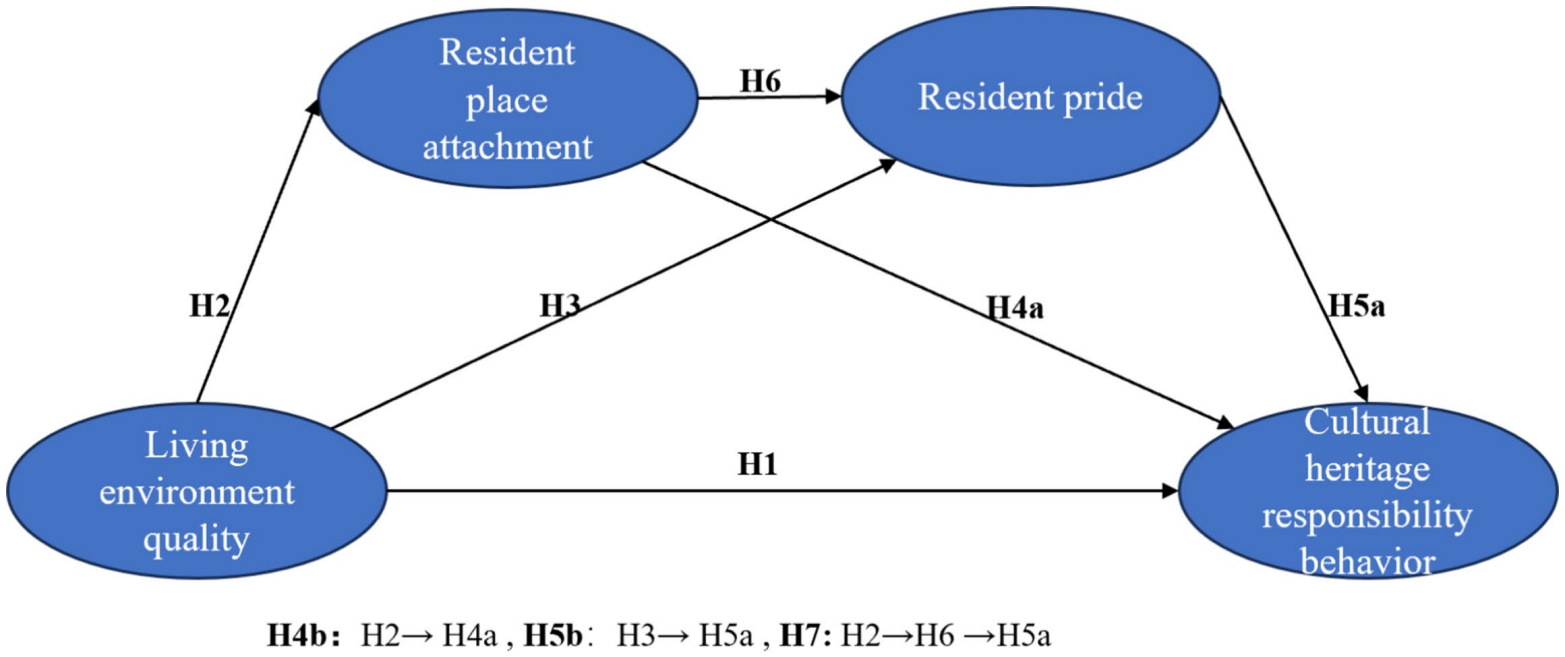
Conceptual model of this research.

Building on the discussions presented in the introduction and literature review, this research investigates how living environment quality influences residents’ responsibility behaviors for cultural heritage through place attachment and pride. The conceptual model is illustrated in Figure 1.

## 3 Method

### 3.1 Study site

**Figure 2:**
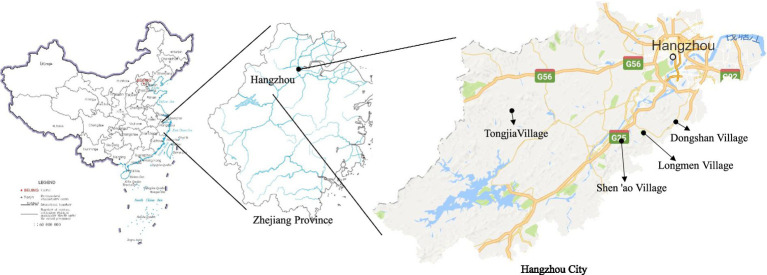
The geographical location of the study sites in Hangzhou, Zhejiang Province, China.

The case study is set in Hangzhou, Zhejiang Province, China, a city renowned for its historical and cultural heritage. Hangzhou is home to a significant number of traditional villages and historical structures in its rural areas, with a total of 166 traditional villages. Among these villages, 65 are designated at the national level, 63 at the provincial level, and 38 at the municipal level. Notably, the national-level traditional villages account for 9.27% of the province’s total of 701 traditional Chinese villages (Figure 2).

To strengthen the protection and development of traditional villages and cultural heritage sites, Zhejiang Province issued the *Guiding Opinions on Strengthening the Protection and Development of Traditional Villages* in 2016, providing institutional support for conservation efforts. The Hangzhou government has prioritized preserving traditional villages and rural cultural heritage by fully implementing protective measures for historic structures, accelerating restoration efforts, and effectively preventing the deterioration of ancient buildings and dwellings. At the same time, the government addresses villagers’ needs for modern production and living by improving village infrastructure and expediting the development of public service facilities (Wang et al., 2023).

Research on traditional villages in Hangzhou not only enhances the precision and effectiveness of rural revitalization and cultural heritage protection but also helps establish clear priorities for their protection and development. It refines developmental positioning, strengthens cultural inheritance efforts, and provides a scientific foundation for the sustainable development of traditional villages in other regions.

From the national-level traditional villages in Hangzhou, four villages—Tongjia, Shen’ao, Longmen, and Dongshan—were selected for field investigation using a convenient sampling method. The basic information and cultural heritage status of these villages are presented in Table 1. These four villages exhibit certain variations in topography, population size, tourism development level, dominant industries, and types of cultural heritage. Such internal diversity enhances the sample heterogeneity to some extent, facilitating the capture of distinct characteristics across traditional villages and thereby reducing sample bias. Nevertheless, it should be acknowledged that Hangzhou’s traditional villages possess advantageous positions in terms of resource endowment and economic foundation, making them less representative of traditional villages in regions with scarce cultural resources or weaker policy support. Consequently, the generalizability of this study’s findings to areas with limited resources or inadequate policy support warrants caution.

**Table 1:** Basic information of selected villages.

Basic situation	Tongjia village	Shen’ao village	Longmen village	Dongshan village
Village area (sq. km)	17.774	5.192	3.47	3.69
Population structure	377 households, 1,228 people	1,290 households, 4,262 people	566 households, 1,879 people	318 households, 1,044 people

Terrain and relief	Mountainous region	Hill	Hill	Mountainous region
Representative cultural heritage	The *He Clan Ancestral Hall* has been designated as a municipal-level cultural heritage protection unit. Several intangible cultural heritage projects are associated with this area, including the *Hongling Mountain Mantou Festival*, the *He Family Qingmao Lion Dance*, and the *Zhang Family Paper Bamboo Dragon Lantern*	The region preserves over 100 traditional buildings, covering approximately 40,000 sq. m, with more than 40 Qing Dynasty structures. Intangible heritage projects here also include the making of *Shen’ao Colored Lanterns*, *Mi Guo Rice Cakes*, and *Lion Dance Performances*.	This region boasts an extensive collection of ancient Ming and Qing architecture, such as ancestral halls, townhouses, residences, ancient towers, stone bridges, and archways. Intangible cultural practices such as the *Longmen Horse Battle* and *Jumping Kui Star* performances also thrive here.	The area contains five ancient buildings over 100 years old and 56 traditional architectural sites, alongside unique traditions such as the *Dongshan Rice Cake Festival, Traditional Moonshine Brewing,* and *the Killing of New Year Pigs Festival.*

### 3.2 Measurement

The questionnaires used in this study employed a 5-point Likert scale, where 1 represented strong disagreement and 5 represented strong agreement. The measurement items for living environment quality in the questionnaire were adapted from the wording used by Hu and Wang (2020) and Wang and Zhu (2022). The place attachment items were derived from established scales developed by Raymond et al. (2010) and Rollero and De Piccoli (2010). Pride was measured using adapted items from the validated scales of Soulard et al. (2024) and Cao et al. (2024). The measurement of residents’ cultural heritage responsibility behaviors was adapted from the established scales of Rasoolimanesh et al. (2017) and Megeirhi et al. (2020), with three items specifically capturing “active participation,” “negative deterrence,” and “cognitive enhancement,” thereby covering different dimensions of heritage responsibility behaviors and ensuring conceptual measurement comprehensiveness.

In addition, the questionnaire included questions to collect demographic information such as gender, age, education level, marital status, and family status. As some measurement items in the questionnaire were originally in English, the research team collaborated with native English-speaking researchers to translate these items, ensuring accuracy and clarity in the research content.

### 3.3 Data collection

This research employed a questionnaire survey, with questionnaires randomly distributed to residents of four traditional villages: Tongjia, Shen’ao, Longmen, and Dongshan. To ensure the reliability and validity of the questionnaire, a pilot survey was performed prior to the main data collection. A total of 80 pilot questionnaires were distributed among residents at the study sites. Preliminary analysis revealed a standardized Cronbach’s *α* coefficient of 0.958, surpassing the threshold of 0.8 and indicating a high level of reliability for the questionnaire. The formal survey was conducted between July 2024 and October 2024, during which 446 questionnaires were collected. Of these, 425 were deemed valid, yielding an effective response rate of 95.29%. The demographic details of the participants are presented in Table 2.

**Table 2:** Profile of the survey participants.

Variable	Category	*n*	%
Sex	Male	237	55.76
	Female	188	44.24
Age	<18	20	4.71
	18–30	64	15.06
	31—40	92	21.65
	41—50	91	21.41
	51—60	102	24.00
	>60	56	13.18
Education	High school and below	262	61.65
	Junior college	73	17.18
	Undergraduate course	78	18.35
	Postgraduate and above	12	2.82

*n* = 425.

## 4 Results

### 4.1 Measurement properties

In this study, SPSS 25.0 and AMOS 24.0 were used for data analysis and model evaluation. First, skewness and kurtosis tests were conducted to assess the normality of the sample data. The skewness values ranged from −0.247 to −0.857, and the kurtosis values ranged from −0.931 to 0.339, indicating that the data followed a normal distribution (Joanes and Gill, 1998). Second, a prior sample size calculation was performed to determine the number of participants required for the model (Rao, 1977). Confirmatory factor analysis (CFA) was conducted on 7 factors and 21 analysis items using an effective sample size of 425, which exceeded the minimum required ratio of 10 participants per analysis item, signifying a moderate sample size.

Confirmatory factor analysis (CFA) was performed to assess whether the correspondence between measurement factors and items aligned with the researcher’s expectations, thereby ensuring the validity and reliability of the research data (Hurley et al., 1997). The model fitting indices were as follows: χ^2^/df = 2.208, GFI = 0.922, RMSEA = 0.053, RMR = 0.036, CFI = 0.969, NFI = 0.946, and NNFI = 0.962—all within acceptable thresholds. The standardized estimates for all measurement relationships exceeded 0.6 and were statistically significant, indicating a strong measurement relationship (Gagné and Hancock, 2006). The average variance extracted (AVE) values for all seven factors were greater than 0.5, while the composite reliability (CR) values exceeded 0.7, demonstrating excellent convergent validity, as shown in Table 3. Furthermore, discriminant validity was confirmed, as outlined in Table 4. The square root of the AVE for each factor was greater than the absolute value of its correlation coefficient with other factors, indicating good discriminant validity.

**Table 3:** Confirmatory factor analysis results.

Constructs and Items	Factor Loading	Cronbach’s *α*	AVE	CR
Natural environment		0.897	0.742	0.896
Climatic environment	0.851			
Green coverage rate of vegetation	0.856			
Water resources such as rivers and ponds	0.877			
Hardware facility		0.840	0.638	0.841
Cultural, sports, and recreational facilities	0.767			
Environmental sanitation facility	0.830			
Basic living facilities	0.799			
Social governance		0.919	0.796	0.921
Layout, construction, and planning	0.890			
People and neighbors	0.904			
Social security order	0.883			
Housing condition		0.833	0.627	0.835
The structural design of the house	0.788			
The space area of the house	0.802			
Landscape design inside and outside the house and yard	0.782			
Place attachment		0.863	0.679	0.864
I like our village best	0.824			
Our village is irreplaceable to me	0.843			
I have a strong sense of identity with our village	0.804			
Pride		0.840	0.639	0.841
The development of the village has improved the living standards of the villagers and made me feel proud	0.842			
The village is a traditional Chinese village, which makes me proud	0.791			
I am proud of the future prospects of the village	0.762			
Responsibility behaviors of cultural heritage		0.848	0.653	0.850
Participate in various cultural heritage protection activities in the village	0.788			
Discourage and stop acts of damage to cultural heritage	0.823			
Take the initiative to learn about cultural heritage	0.813			

**Table 4:** Discriminative validity: Pearson’s correlation and AVE square root values.

Dimension	Natural environment	Hardware facility	Social governance	Housing condition	Place attachment	Pride	Responsibility behaviors of cultural heritage
Natural environment	**0.861**						
Hardware facility	0.631	**0.799**					
Social governance	0.663	0.696	**0.892**				
Housing condition	0.618	0.636	0.727	**0.792**			
Place attachment	0.579	0.647	0.656	0.615	**0.824**		
Pride	0.604	0.684	0.653	0.625	0.698	**0.799**	
Responsibility behaviors of cultural heritage	0.536	0.641	0.598	0.574	0.733	0.729	**0.808**

Diagonal bold numbers are AVE square root values.

### 4.2 Assessment of the structural model

**Figure 3:**
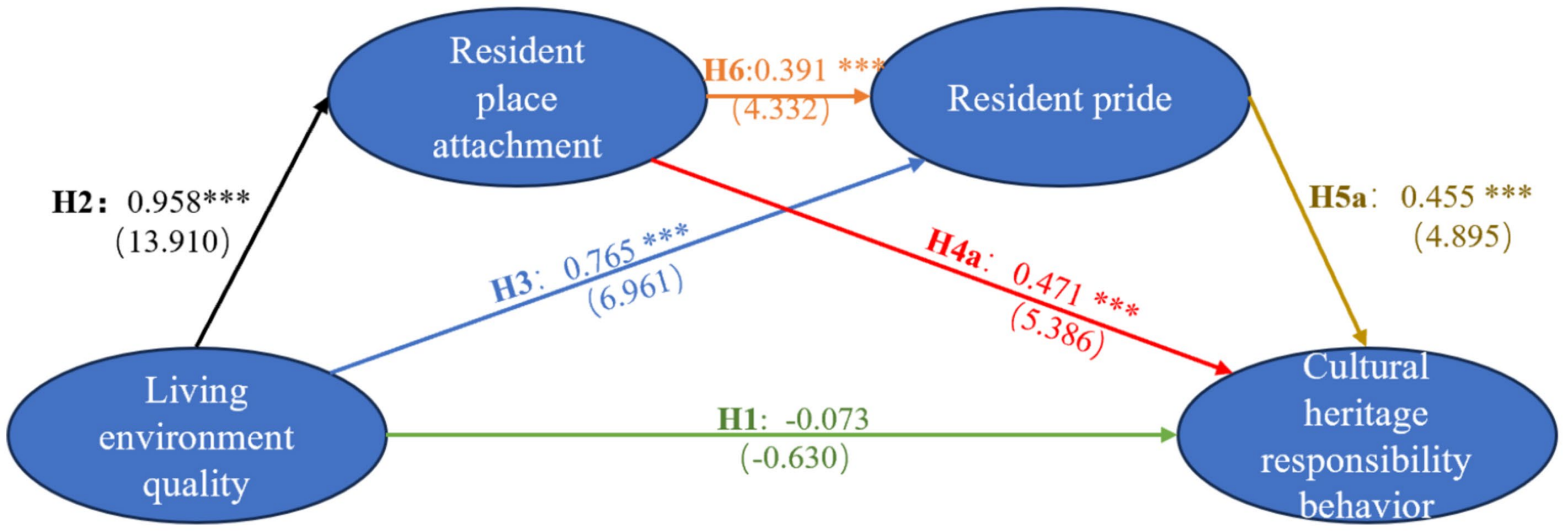
AMOS output results of the proposed model.

The structural equation model was constructed in AMOS, and the model fit indices were as follows: χ^2^/df = 2.182, GFI = 0.918, RMSEA = 0.053, RMR = 0.040, CFI = 0.968, and NFI = 0.943. All indices fell within acceptable ranges, indicating a high level of model fit (Figure 3 and Table 5).

**Table 5:** Structural model assessment and hypotheses test results.

Hypotheses	Paths	Standardized coefficient	t-value	Results
H1	Living environment quality→ Cultural heritage responsibility behavior	−0.073	−0.630	Not supported
H2	Living environment quality→ Place attachment	0.958	13.910***	Supported
H3	Living environment quality→ Pride	0.765	6.961***	Supported
H4a	Place attachment →Cultural heritage responsibility behavior	0.471	5.386***	Supported
H5a	Pride →Cultural heritage responsibility behavior	0.455	4.895***	Supported
H6	Living environment → Pride	0.391	4.332***	Supported

*p < 0.05; **p < 0.01; ***p < 0.001.

The path coefficient between living environment quality and responsibility behavior was 0.116 (*p* > 0.05), indicating that H1 is not supported. However, significant relationships were observed for living environment quality and place attachment (*β* = 0.069, *p* < 0.001), place attachment and cultural heritage responsibility behavior (β = 0.088, *p* < 0.001), living environment quality and pride (β = 0.110, *p* < 0.001), pride and cultural heritage responsibility behavior (β = 0.093, *p* < 0.001), and place attachment and pride (β = 0.090, *p* < 0.001). These results indicate that H2, H3, H4a, H5a, and H6 are supported.

### 4.3 Assessment of mediation effects

The estimator used in intermediary analysis is highly sensitive to deviations from the assumption of normality. To ensure the reliability of the intermediary effect, the bootstrapping method was employed (Alfons et al., 2022). This approach empirically tested sensitive data characteristics, such as outliers, heavy tails, and skewness in the observed distribution, which could potentially undermine the validity of the mediation mechanism.

In Table 6, the total effect of living environment quality on cultural heritage responsibility behavior [95% CI (0.752, 1.047)], the indirect effect of living environment quality on cultural heritage responsibility behavior through place attachment [95% CI (0.240, 0.687)], and the indirect effect of living environment quality on cultural heritage responsibility behavior [95% CI (0.240, 0.687)] were presented. The indirect impact of living environment quality on cultural heritage responsibility behavior through pride [95% CI (0.179, 0.590)], the impact of living environment quality on cultural heritage responsibility behavior through place attachment and pride in turn [95% CI (0.073, 0.324)], and their confidence intervals do not include zero values. Therefore, the intermediary effect is significant. However, the confidence interval of the direct impact of the quality of living environment on the responsibility behavior of cultural heritage [95% CI (−0.368, 0.188)] contains zero values. Therefore, the direct impact is not significant, and this mediation effect is judged to be completely mediating. Therefore, H4b, H5b, and H7 were supported.

**Table 6:** Path mediation results.

Paths	Estimation	Bootstrapping 5,000		*p* value
		Percentile 95% CI		
		Lower	Upper	
Total effect				
Living environment quality → Cultural heritage responsibility behavior	0.897	0.752	1.047	0.001
Direct effect				
Living environment quality → Cultural heritage responsibility behavior	−0.073	−0.368	0.188	0.608
Indirect effect				
H4b: Living environment quality → Place attachment → Cultural heritage responsibility behavior	0.451	0.240	0.687	0.001
H5b: Living environment quality → Pride → Cultural heritage responsibility behavior	0.348	0.179	0.590	0.001
H7: Living environment quality → Place attachment → Pride → Cultural heritage responsibility behavior	0.171	0.073	0.324	0.001

## 5 Discussion

### 5.1 Conclusion

Based on empirical analysis and the PAD temperament model, this study examines the relationships between living environment quality, residents’ place attachment, pride, and cultural heritage responsibility behaviors, leading to the following conclusion.

First, the findings reveal that the quality of the living environment in traditional villages directly and positively influences residents’ place attachment and pride (i.e., H2 and H3 are confirmed). As previous hospitality and tourism studies indicate, place attachment, as a psychological bond between residents and their communities, is deeply rooted in specific locations and evolves over time through continuous interactions between individuals and their surrounding environment, which both shapes and is shaped by this relationship (Jorgensen and Stedman, 2001). Similarly, pride represents a form of identity grounded in social belonging. Residents’ experiences in public living spaces enhance their sense of belonging and inclusion within the community (Williams and DeSteno, 2008). In line with these prior findings, this research confirms that traditional village living environments serve as architectural, social, and relational spaces where daily interactions foster residents’ sense of belonging, identity, and strengthened place attachment and pride among residents.

Second, our results indicate that place attachment and pride directly and positively influence residents’ cultural heritage responsibility behaviors (supporting H4a and H5a). This is consistent with previous findings which confirm the significant impact of emotional attitudes on behavioral patterns (Shin and Yang, 2022). As emotional attitudes, place attachment and pride directly motivate residents to engage in prosocial activities. For instance, PA and pride have been confirmed to individually affect residents’ positive environmental behaviors (Buta et al., 2014; Sadler and Pruett, 2017). Community residents’ active participation is also essential for effective cultural heritage protection (Qin and Leung, 2021). Our empirical results suggest that the psychological bond formed through residents’ interaction with traditional village spaces integrating cultural elements with personal experiences, fostering recognition of the villages’ value, and encouraging behaviors that support cultural heritage conservation.

Third, the research findings indicate that the impact of traditional village living environment quality on residents’ cultural heritage responsibility behavior, mediated by place attachment and pride, follows a complete mediation model. This implies that the total effect of the living environment on cultural heritage responsibility behavior is entirely transmitted through place attachment and pride. When the intermediary variables are introduced into the model, the influence of the independent variable on the dependent variable is entirely absorbed by these intermediary variables, leaving no direct independent effect (Maxwell et al., 2011). This finding shows that the quality of living environment quality does not directly affect the residents’ cultural heritage responsibility behavior but indirectly affects their responsibility behavior through their attachment and pride to traditional villages. In addition, the causal and sequential relationship between the quality of living environment → place attachment → pride → residents’ cultural heritage responsibility behavior shows the complex influence between the quality of traditional village living environment and residents’ cultural heritage responsibility behavior. This finding reveals that the impact of the quality of living environment on the responsibility behavior of cultural heritage completely depends on the emotional identification of residents, and these emotional connections and identification can be further transformed into practical actions, that is, the responsibility behavior of cultural heritage.

### 5.2 Theoretical implications

The findings of this study provide a new perspective and theoretical basis for understanding and promoting the responsibility behavior of traditional village cultural heritage.

First, our research substantiates the pivotal role that living environment quality plays in promoting the sustainable development of cultural heritage within traditional villages. Previous studies have indicated that heritage protection contributes to community development and environmental enhancement (Elsorady, 2012) and that the human living environment also fosters the sustainable development of villages (Jiang et al., 2022; Wei et al., 2023). However, there remains a scarcity of empirical data and theoretical backing for the direct influence of the human living environment on the sustainable development of cultural heritage in traditional villages. Our findings offer empirical and theoretical validation for the reciprocal relationship between the quality of the living environment and cultural heritage protection. Specifically, we demonstrate that enhancing residents’ living conditions not only supports community development but also actively encourages residents to engage in heritage protection. In essence, we propose a bidirectional dynamic: cultural heritage protection improves the living conditions of community residents, while enhancements in the living environment stimulate cultural heritage protection behaviors.

Second, the study proves the applicability of PAD model in the relationship between the quality of human settlements and residents’ behavior in traditional villages. The quality of village human settlement environment can improve the emotional pleasure of residents (Prezza et al., 2001) and has a significant positive effect on the pride and place attachment as residents’ physiological activation, thus promoting the responsible behavior of individuals for situation control. Within the framework of PAD model, our study verifies the positive impact of the human settlement environment in the traditional village’s physical space and social living space on psychological emotion and individual responsible behavior. This not only expands the applicability of the PAD temperament model but also provides more detailed empirical data supporting the validity of the theory, thereby facilitating its application to a broader range of research areas. In addition, residents’ active participation in cultural heritage activities can also bring multiple benefits. By personally engaging in protection practices, not only can it deepen residents’ understanding of the cultural value of the community and enhance their sense of community belonging but it can also improve self-efficacy and social identity through interaction, thereby directly gaining emotional satisfaction and motivation (Qin and Leung, 2021). Therefore, we can also argue that residents’ participation in cultural heritage activities may similarly strengthen their place attachment and pride.

Third, the study found that place attachment and pride mediated the quality of living environment and the responsibility behavior of cultural heritage. This shows that the improvement of the living environment of traditional villages cannot directly change the behavior of residents. It indirectly affects the behavior of residents through its influence on their emotional attitude. This complete mediation effect provides a new theoretical contribution to the field of environmental psychology and cultural heritage protection and further explains the impact mechanism of rural living environment quality on the sustainable development of cultural heritage.

### 5.3 Practical applications

In addition to the theoretical implications, this research also offers significant practical applications as follows.

First, our findings emphasize the impact of the living environment quality on residents’ place attachment and pride, which are directly linked to their cultural heritage conservation behaviors. Therefore, protection and renewal strategies for traditional villages should prioritize residents’ emotional needs and quality of life (Mu and Aimar, 2022). Practical measures, such as improving infrastructure, optimizing public spaces, and preserving traditional architectural elements, can enhance residents’ sense of attachment and pride. This, in turn, stimulates their active participation in cultural heritage protection and promotes sustainable community development.

Second, the research shows that local attachment and pride play a completely mediating role in the quality of the living environment of traditional villages and the responsibility behavior of residents’ cultural heritage. Improving the village living environment should focus on fostering positive emotional connections among residents, empowering them to play a central role in enhancing their surroundings. Active resident participation in environmental improvements, coupled with attention to subjective emotional experiences, can strengthen community identity and a sense of belonging (Jing et al., 2024). For example, revitalization efforts could include converting traditional buildings into homestays or craft exhibition halls, thereby achieving dual goals: improving residents’ living conditions and ensuring cultural heritage protection. This approach not only supports local economic growth but also sustains cultural heritage conservation.

Finally, our findings provide a scientific basis for planning and policy formulation to support the sustainable development of traditional villages. Policymakers and planners should consider the interplay between the living environment quality and residents’ cultural heritage conservation behaviors. Development plans should integrate quality-of-life improvements with heritage protection initiatives. Establishing relevant policies can encourage collaboration among governments, community, and residents, forming a sustainable mechanism for cultural heritage preservation. This inclusive approach ensures long-term engagement from all stakeholders in protecting the unique cultural assets of traditional villages.

### 5.4 Limitations and future research

Similar to other studies, this research has a few limitations. First, the sample may not adequately capture the diversity of traditional villages and their populations. To enhance the universality and precision of future studies, researchers should expand the sample scope to include a wider range of traditional villages and diverse resident groups. This approach will provide a more comprehensive understanding of how living environment quality impacts cultural heritage preservation. Second, the analysis of place attachment and pride as mediators may overlook other potential mediating or moderating variables that merit further exploration. Considering these limitations, future research should explore residents’ emotional experiences and behavioral motivations through case studies, interviews, and qualitative methods to better capture the complexities of mediating effects. Finally, cross-cultural and cross-border comparisons should also be considered to account for the diversity of traditional villages and their living environments, offering broader insights into the factors influencing cultural heritage responsibility behaviors.

## Data Availability

The raw data supporting the conclusions of this article will be made available by the authors, without undue reservation.
